# A novel method to identify and characterise peptide mimotopes of heat shock protein 70-associated antigens

**DOI:** 10.1186/1476-8518-4-2

**Published:** 2006-04-08

**Authors:** Blanca Arnaiz, Laura Madrigal-Estebas, Stephen Todryk, Tharappel C James, Derek G Doherty, Ursula Bond

**Affiliations:** 1 Moyne Institute for Preventive Medicine, Department of Microbiology, University of Dublin, Trinity College, Dublin 2, Ireland; 2 Institute of Immunology & Department of Biology, National University of Ireland, Maynooth, Co. Kildare, Ireland; 3 Centre for Clinical Vaccinology and Tropical Medicine, Churchill Hospital, Oxford OX3 7LJ, UK

## Abstract

The heat shock protein, Hsp70, has been shown to play an important role in tumour immunity. Vaccination with Hsp70-peptide complexes (Hsp70-PCs), isolated from autologous tumour cells, can induce protective immune responses. We have developed a novel method to identify synthetic mimic peptides of Hsp70-PCs and to test their ability to activate T-cells. Peptides (referred to as "recognisers") that bind to Hsp70-PCs from the human breast carcinoma cell line, MDA-MB-231, were identified by bio-panning a random peptide M13 phage display library. Synthetic recogniser peptides were subsequently used as bait in a reverse bio-panning experiment to identify potential Hsp70-PC mimic peptides. The ability of the recogniser and mimic peptides to prime human lymphocyte responses against tumour cell antigens was tested by stimulating lymphocytes with autologous peptide-loaded monocyte-derived dendritic cells (DCs). Priming and subsequent stimulation with either the recogniser or mimic peptide resulted in interferon-γ (IFN-γ) secretion by the lymphocytes. Furthermore, DCs loaded with Hsp70, Hsp70-PC or the recogniser or the mimic peptide primed the lymphocytes to respond to soluble extracts from breast cells. These results highlight the potential application of synthetic peptide-mimics of Hsp70-PCs, as modulators of the immune response against tumours.

## Background

Both T- and B-cell immune responses to tumour-derived proteins have been identified in many cancer patients, however the responses are generally insufficient to result in tumour clearance. One of the challenges in cancer treatment is to enhance this anti-tumour immune response, perhaps by identifying novel tumour antigens with a higher immunogenic potential. Such antigens have the potential to be tumour biomarkers in serological testing and targets in anti-tumour vaccine development. Currently there are many serologically defined protein tumour markers known and in some cases the corresponding peptide sequences have been identified [1]. Promising results have been observed following vaccination with antigenic peptides derived from the 'cancer-testis' antigen, MAGE-3, NY-ESO-1 and the melanocyte differentiation antigens Melan-A/MART-1/tyrosinase and gp100 [2,3].

Tumour-derived heat shock protein (Hsp) preparations have been shown to elicit anti-tumour immune responses in both mice and man [4]. In mice, immunisation with tumour cell extracts was shown to confer immuno-protection against a subsequent challenge with the same tumour. When these extracts were fractionated, the stress proteins, Hsp70 and gp96 were identified as the protective agents [5-7]. Further experiments showed that it is the peptides complexed with these proteins that are responsible for the generation of tumour-specific immune responses [8-11].

Recent studies have shown that chaperones such as heat shock proteins gp96, Hsp90, Hsp70 and calreticulin can be taken up by dendritic cells by receptor mediated endocytosis, where they enter the MHC class I antigen presentation pathway and are cross-presented to T-cells [12-19]. Additionally, both gp96 and highly purified Hsp70 have been shown to directly stimulate monocytes and dendritic cells to secrete cytokines, in a manner similar to LPS. They also up-regulate HLA and other co-stimulatory molecules, thereby enhancing the presentation of any associated chaperoned peptides to the T-cells [20]. This dual function of 'adjuvant-cum-antigen pool', make gp96- and Hsp70-peptide complexes, (referred to as gp96-PCs and Hsp70-PCs), good candidates for tumour vaccines. In this regard, some very exciting and crucial clinical trials to stimulate immune responses using autologous gp96-PCs and Hsp70-PCs purified from resected tumours are ongoing with some encouraging outcomes in patients with melanoma [21]. Autologous gp96-PCs are currently being tested for the treatment of lymphoma, renal cell carcinoma, colorectal, gastric, pancreatic and breast cancers while Hsp70-PCs are being tested for the treatment of chronic myelogenous leukemia (CML; Antigenics Inc., New York, NY).

A novel approach to the development of tumour vaccines has been the isolation of peptide mimics to epitopes of known oncogene products or tumour specific antigens. A classical example of this is the anti-idiotype antibody 105AD7 which inhibits the binding of the monoclonal antibody 791T/36 to its antigen TAA gp72 [22,23]. Subsequent studies have revealed that 105AD7 mimics the epitope of a widely expressed cellular protein CD55 [24]. A large number of anti-idiotype antibodies have been identified and many have been used with or without modifications in cancer immunotherapy [25-27]. Bio-panning of peptide phage display libraries using antibodies to known tumour antigens have led to the identification of mimic epitopes (mimotopes) [28-34]. Such selected 'mimotopes' can elicit highly specific humoral immune responses against the peptides and/or the original tumour antigen. Although the baits used in the above studies were relatively pure, it is now well established that successive rounds of rigorous bio-panning will select/enrich and amplify ligands even from a mixture of baits. For example, intravenously administered phage display library has been successfully used for *in vivo *bio-panning in certain animal model systems to identify tissue-specific peptide ligands [35,36] and see refs. [37,38] for reviews.

We have devised a novel approach to generate peptides that mimic the antigenicity of tumour cell-derived Hsp70-PCs, first by screening a random peptide M13 phage display library using as bait Hsp70-PCs extracted from the human breast cancer cell line, MDA-MB-231 to identify putative Hsp70-PC binding phages (recogniser phages). After several rounds of bio-panning, a number of 'recogniser peptides' were identified. Subsequently, we used selected synthetic 'recogniser peptides' as baits in a 'reverse' bio-panning experiment to identify phages that interact with the recogniser peptides. Our hypothesis suggests that such phages may display peptides that are putative structural mimics of the Hsp70-PCs. One of the 'recogniser peptides' used in the reverse bio-panning led to the enrichment of a single class of phages all coding for the same mimic peptide. We tested the ability of this mimic peptide to stimulate human lymphocytes, either directly or presented by autologous monocyte-derived dendritic cells (DCs). Our results show that CD14^- ^PBMCs primed with the mimic peptide loaded onto DCs, produce IFN-γ upon a second stimulation with the same peptide. Furthermore, CD14^- ^PBMCs primed with DCs loaded with Hsp70-PC from MDA-MB-231 cells *in vitro*, produced IFN-γ upon subsequent stimulation with the mimic peptide. Our results also show that CD14^- ^PBMCs primed with DCs loaded with the mimic peptide, produce IFN-γ when challenged with soluble cell extracts from either MDA-MB-231 (tumourigenic) or MCF-12A (non-tumourigenic) breast cell lines. These results suggest that the synthetic mimic peptide immunologically resembles peptides present in the protein extracts from breast cell lines and more specifically resembles peptides complexed with Hsp70. Thus, the approach outlined in this paper for the detection of Hsp70-PC mimics should prove extremely useful in the identification of tumour-specific peptide mimics with immune modulatory properties.

## Materials and methods

### Cell lines

The breast cancer cell line MDA-MB-231 was a generous gift from Dr. Boucher-Hayes, Beaumont Hospital, Dublin, and was grown in RPMI-1640 supplemented with 10% foetal calf serum (FCS). The normal breast cell line MCF-12A was purchased from ATTC-LGC (Teddington, U.K) and grown in supplemented DMEM as recommended by the supplier. Media also contained 2 mM L-glutamine, 1× antibiotic/antimycotic solution (Sigma Chemical Co.) and 100 U/mL nystatin suspension.

### Cell extracts and purification of Hsp70 and Hsp70-PCs

To prepare tumour cell extracts, the MDA-MB-231 or MCF-12A cells were trypsinised and harvested. Cells were washed twice in ice-cold PBS and the cell pellets resuspended in 1 mL PBS and lysed by 5 freeze/thaw cycles followed by sonication. The insoluble material was pelleted by centrifugation (20,500 × g for 30 min.). The supernatants were aliquoted and stored at -20°C. The Hsp70 and Hsp70-PCs were purified from 10^8 ^MDA-MB-231 or MCF-12A cells as previously described [7]. The purified proteins were analysed by SDS-PAGE, and immunoblotting using mouse monoclonal anti-Hsp70 and biotinylated anti-mouse secondary antibodies (Sigma Chemical Co.) in a streptavidin-horseradish peroxidase based chemiluminescence detection system.

### Bio panning and library amplification and selection

Approximately 10^11 ^phage particles from a 12-mer M13 phage display library (PHD-12; New England Biolabs Inc., MA) were used for each bio-panning experiment. Approximately 10 μgs of Hsp70-PCs (100 μg/mL) were immobilised in a single well of a 96-well Maxisorb (Nunc-Nalge Inc.) microtitre plates. The blocking, binding and washing strategies were carried out as instructed by the manufacturer with the following exceptions. We used (a) either 1% Bovine Serum Albumin (BSA) or casein for blocking non-specific binding alternating these blocking substrates between subsequent rounds of bio-panning to prevent selection of phage recognising the blocking substances, (b) competitive elution with the bait Hsp70-PCs in rounds three and four and (c) bio-panning in solution for rounds two and four, using biotinylated Hsp70-PCs and a streptavidin matrix to prevent selection of plastic-binding phages. In the latter case, 10 μg of Hsp70-PCs were biotinylated using NHS-Biotin (Sigma Chemical Co., Poole, U.K.) according to the manufacturer's instructions and incubated with 10^11 ^phage particles as recommended by the manufacturer with either of the blocking reagents. The Hsp70-PCs bound phage particles were recovered either through a Streptavidin-agarose (Pierce Chemical Co. ILL) column or Streptavidin-Dynabeads (Dynal Co., Norway) followed by washing and competitive elution as described above. Following each round of bio-panning, the eluted phages were amplified to high titer according to supplier's instructions. A subtraction screening using the peptide depleted Hsp70-PC [7] was performed after the third bio-panning to remove those phages recognising the Hsp70 portion of the Hsp70-PC bait. The unbound fraction was amplified and used in the fourth round bio-panning. The phage particles from the final fourth round eluate were plated at low density to allow isolation of single phage clones. The DNA insert from the amplified phage clones was sequenced and the Hsp70-PCs binding 12-mer recogniser peptide sequences were deduced. To identify potential Hsp70-PCs mimic peptides, the recogniser peptides (including the tri-Glycine linker) were synthesised (see below) and used as bait in a similar bio-panning procedure as above with the exception that the subtraction step with Hsp70 was not included. Special attention was taken to ensure the binding of the bait peptide to the matrix. After 4 rounds of bio-panning, the eluted phage displaying mimic peptides were analysed as above.

### Peptides

The C-terminal amidated peptides, TMG (recogniser), DSP (mimic), and WHK (mimic), with a three glycine-spacer arm and with or without an N'-terminal biotin tag were synthesized at the Advanced Biotechnology Centre (Imperial College, U.K).

### M13 phage ELISA

Biotinylated DSP or TMG peptides (200 pmoles) were bound to 200 mg streptavidin-coated magnetic beads (Dynal Co, Norway) according to manufacturer's instructions. Following blocking with 0.5% BSA and 0.1 mM D-biotin in PBS, the beads were incubated for three hours with selected M13 phage clones (10^11 ^pfu) displaying either the TMG or DSP peptides. Unbound phages were removed by repeated washing with excess PBS containing 0.05% TWEEN-20 (PBS-Tween). The bound phages were detected by incubation with HRP conjugated anti-M13 monoclonal antibody (anti-M13-HRP; Amersham Biosciences, UK; 1:2,500) according to suppliers instructions except that the beads were transferred to wells of a microtiter plate prior to colour development. As controls, beads alone without peptides were processed through the same procedure.

### Isolation of monocytes and lymphocytes and generation of immature DCs

Buffy coat packs from healthy female donors were obtained from the Irish Blood Transfusion Service. PBMCs were prepared by Lymphoprep (Nycomed, Oslo, Norway) density gradient centrifugation. Monocytes were isolated by positive selection of CD14^+ ^cells using CD14 Microbeads (Miltenyi Biotec, Bergisch Gladbach, Germany). Immature DCs (iDCs) were generated by culturing monocytes for 6 days in RPMI-1640 medium supplemented with 10% endotoxin-free foetal calf serum, 2 mM L-glutamine, 80 U/mL each of penicillin and streptomycin, 2 μg/mL amphotericin B in the presence of 60 ng/mL recombinant human granulocyte macrophage colony stimulating factor (GM-CSF) and 150 ng/mL recombinant human IL-4. Medium and cytokines were replaced every 2 days. The CD14^- ^PBMC, which mainly consist of lymphocytes (B cells, T cells, NK cells and NKT cells) were cryopreserved for later use as responder cells.

### *In vitro *stimulation assay of lymphocytes

Lymphocyte stimulation was performed using a modification of a published procedure [39]. Approximately 10^4 ^immature dendritic cells (iDCs) were incubated with 100 μl of medium containing 10 μg/mL LPS and peptides (25 μg/mL DSP, TMG or WHK), MDA-231 or MCF-12A cell extracts (total protein concentration 110 μg/mL in each case), or Hsp70 or Hsp70-PCs (both at 5 μg/mL) or media alone. After 24 hour incubation, the cells were pelleted and the culture media were assayed for IL-12 by ELISA (see below). The iDCs were resuspended in RPMI, γ-irradiated with a dose of 5,000 rads and washed in RPMI. The irradiated iDCs were incubated with CD14^- ^PBMCs at a 1:10 ratio in a final volume of 100 μL of supplemented RPMI. After 48 hours incubation, the supernatants were assayed for IFN-γ by ELISA. These cells (designated as 'primed CD14^- ^PBMCs) were cultured for a further 10 days in 200 μL of the same media supplemented with human recombinant IL-2 (25 ng/mL) and with media changes every 3 days. On day 9, a new batch of iDCs from the same donor was incubated with peptides, cell extracts or media alone but in the absence of LPS. After 48 hrs, the culture supernatants were assayed for IL-12 levels. Following γ-irradiation and washes, these loaded iDCs were incubated with the primed CD14^- ^PBMCs at a ratio of 1:10 (iDC:CD14^- ^PBMCs). After 2 days incubation the culture supernatants were assayed for IFN-γ content by ELISA.

### Measurement of cytokine release

The IFN-γ released by the stimulated CD14^-^PBMC were measured by ELISA using antibody pairs (DuoSet human IFN-γ; R & D Systems, Oxon, UK). IL-12p40 production by iDCs was detected using DuoSet human IL-12p40 antibodies, R&D Systems).

## Results

### Identification of recogniser and mimic peptides of Hsp70-PCs through bio-panning of an M13 phage display library

**Figure 1:**
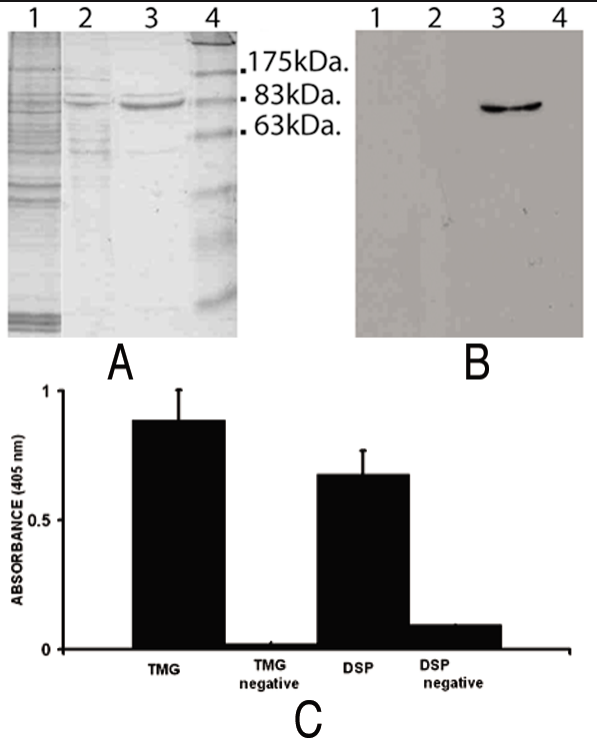
**Purification of Hsp70 and Hsp70-PCs from MDA-MB-231 cells by affinity chromatography**. Hsp70-peptide complexes (Hsp70-PCs) were isolated from whole cell extracts of MDA-MB-231 cells using ADP-Agarose. **A**. Coomassie-Blue stained SDS-polyacrylamide gel and **B**: Western blot using anti-Hsp70 antibody. Lane 1: MDA-MB-231 total cell extract (10 μg), Lane 2: Flow-through from an ADP-agarose column (2 μg), Lane 3: Proteins eluted from ADP-agarose column with 3 mM ADP (2 μg). Lane 4: Molecular weight markers. **C**: ELISA to detect the interaction between biotinylated TMG and DSP peptides and the corresponding phages. Streptavidin-coated paramagnetic beads bound to biotinylated TMG peptide (TMG) or DSP peptide (DSP) were incubated with the M13 phage clones displaying DSP or TMG respectively. As a control, streptavidin-coated beads without the peptides were incubated with M13 phage clone displaying the TMG (TMG negative) or the DSP (DSP negative) peptides alone. All beads were then incubated with anti-M13-HRP antibody. Interactions were detected by absorbance at 405 nm using DAB as a substrate.

**Figure 2:**
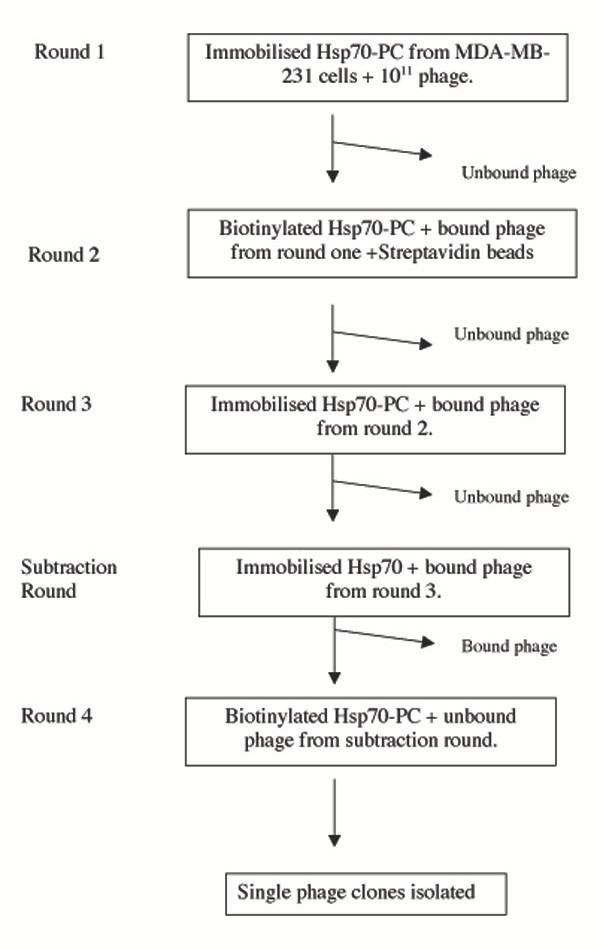
Schematic outline of bio-panning procedure.

Protein fractions enriched in Hsp70-PCs were obtained from MDA-MB-231 cells using ADP-agarose affinity chromatography. Western blot analysis using anti-Hsp70 antibodies and Coomassie Blue staining of the corresponding SDS gel show that the most prominent protein eluted from the column with ADP is the constitutive (Hsp73) and/or the inducible (Hsp72) forms of Hsp70 (Fig. 1A and 1B, lane 3) confirming similar findings by others [7]. To isolate peptides that "recognise" Hsp70-PCs, the column eluate fraction was used as bait to biopan a random peptide M13 phage display library. Four rounds of bio-panning were performed as described in the *Materials and Methods *section and as outlined in Figure 2. Three separate bio-panning experiments were performed using Hsp70-PCs as bait. In each case, approximately 400–1000 phages were retained after four rounds of panning. A total of twenty four phage clones were selected at random for further analysis. DNA was isolated from the phages and was sequenced in order to identify the peptide displayed by each phage clone. The peptide sequence in each case was named according to the first three amino acids in their sequence. As shown in Table 1, a wide variety of peptide sequences were identified in each bio-panning experiment, some of which were common in two of the three bio-pannings. In one case, the phage displaying the TMG peptide was selected in all three bio-pannings (Table 1).

**Table 1:** List of Hsp70-PC (MDA-MB-231 cells) recognising phages

**Recogniser Peptide**	***Frequency (% of total phage clones sequenced)***	***No. of times recovered in independent screens***
TMGFTAPREPHY	10	3
IERPLHESVLAT	16	2
NNYDDISLRARP	22	2
AIPNKLNVWPPH	12	1
TGVSWSVAQPSF	8	1
SQELTQRPYKWH	8	1
TPSYINLXDFIA	8	1
GTSTFNSVPVRD	8	1
KLTFLNYAEVLR	8	1

To identify phage that can interact with the recogniser peptides and thus may represent potential structural mimics of the original Hsp70-PCs, a reverse bio-panning was carried out using synthetic recogniser peptides. Peptides containing the sequences represented by the NNY, IER and TMG phages (Table 1) were synthesized and used as baits. Following four rounds of bio-pannings a group of phage clones were recovered and their DNAs were sequenced. The representative peptide sequences in the enriched phage pool are shown in Table 2. Both IER and NNY peptides selected a number of phages with different peptide sequences. Theoretically, these phages should display structures that 'mimic' the bait used to identify the corresponding recogniser peptides. Both IER and NNY bait peptides selected in high proportion a phage displaying the peptide SVS. However, subsequent literature searches revealed that this peptide has previously been identified in bio-panning experiments using unrelated baits [40,41]. Unlike the IER and NNY peptides, the TMG peptide selected a single class of peptide which is designated DSP (Table 2). Since the TMG peptide was selected in all three independent bio-panning experiments and enriched a single potential mimic peptide sequence we focused our subsequent analysis on the TMG/DSP recogniser/mimic pair.

**Table 2:** List of recogniser peptides and corresponding mimic peptides

***Recogniser Peptides***	***Frequency (% total phage clones sequenced)***	**Mimic Peptides**
NNYDDISLRARP	46	SVSVGMKPSPRP
	18	FHSDWPGXTLTW
	9	LHAETRSAMHRT
	9	WKHTSQPPRLIF
	9	KAXTPVQSASNV
	9	RTHDNSWNYTSS

TMGFTAPREPHY	100	DSPQNPKTWKYI

IERPLHESVLAT	32	SVSVGMKPSPRP
	15	GLPPYSPHRLAQ
	15	NFMESLPRLGMH
	15	NAQNYSQQAPRP
	15	HGLHQMSGNTKR
	8	HPHQPIERQTVQ

### The DSP peptide specifically interacts with phages displaying the TMG peptide

The specificity of the interaction between the TMG and DSP peptides was examined by ELISA (see Materials and Methods). As shown in Fig 1C, phages displaying the DSP peptide specifically bind to a synthetic biotinylated TMG peptide (Fig. 1C, TMG). Conversely, phages displaying the TMG peptide bind to a synthetic biotinylated DSP peptide (Fig. 1C, DSP). In the absence of biotinylated peptides, little or no detectable signal was obtained following incubation of either of the phages alone with streptavidin-coated magnetic beads (Fig. 1C, DSP negative, TMG negative). Furthermore, *in situ *histochemical staining revealed that both the TMG and the DSP peptides display cytoplasmic staining in MDA-MB-231 cells suggesting that these peptides recognise and interact with cellular components (data not shown).

### The DSP and TMG peptides have immune stimulatory properties

**Figure 3:**
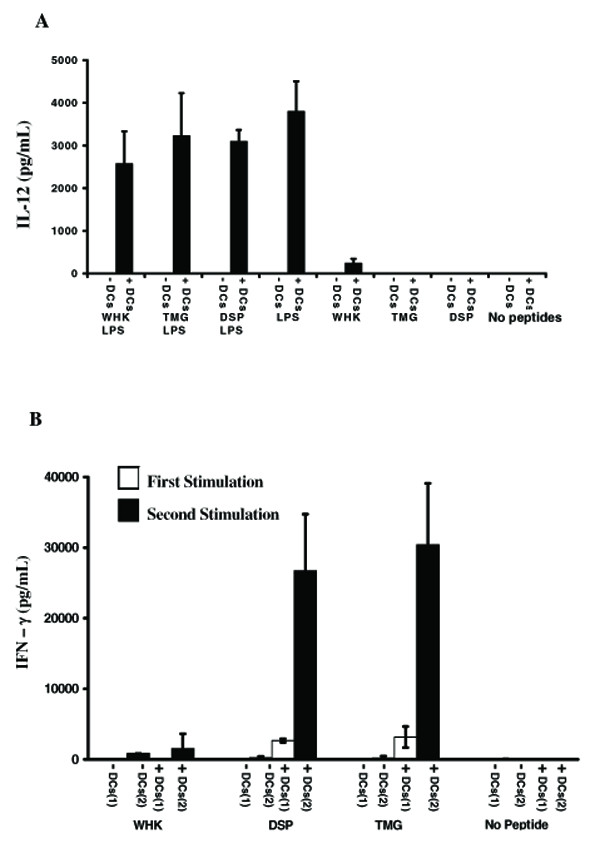
**Recogniser and Mimic peptides presented by iDCs can stimulate CD14^- ^PBMCs to secrete IFN-γ secretion**. **A**. IL-12 production by immature dendritic cells. iDCs (+DCs; black bars) were incubated with or without LPS and either DSP, WHK or TMG peptides for 24 hrs. as labeled. The concentration of IL-12 (pg/mL) in the supernatants was determined by ELISA. IL-12 production in the absence of iDCs was also determined. **B**. iDCs were incubated with WHK, DSP or TMG peptides. Subsequently, either peptide-loaded iDCs (+iDCs) or the free peptide in solution (-iDCs), were incubated with CD14^- ^PBMCs from the same donor [First stimulation; (1) open bars]. These cells were incubated with a second batch of iDCs loaded with the same peptide [Second stimulation (2); filled bars]. The concentration of IFN-γ (pg/mL) in the supernatants of the CD14^- ^PBMCs was determined following the first (1) and second (2) stimulations.

We next examined whether TMG, DSP or an unrelated peptide WHK (Table 2) can stimulate iDCs to release IL-12 and/or lymphocytes to produce IFN-γ. The iDCs were incubated with the peptides DSP, TMG or WHK in the presence or absence of LPS. The culture supernatants were tested for IL-12 by ELISA. Figure 3A shows that IL-12 secretion was detected only when LPS was included but not in its absence when iDCs were stimulated with the peptides alone.

CD14^- ^PBMCs were incubated with TMG, DSP or WHK peptides either directly in solution or after loading onto autologous monocyte-derived iDCs. The supernatants were removed for analysis of IFN-γ production by ELISA after 2 days. These cells were then cultured for a further 10 days in the presence of IL-2, following which they were re-stimulated either with the respective peptide in solution or a second batch of the iDCs from the same donor loaded with the peptides. After a further 2 days the supernatants were tested for the presence IFN-γ. The results (Fig. 3B) show that no IFN-γ was released, either in response to the first or second stimulation with any of the peptides when iDCs were excluded. However, CD14^- ^PBMCs responded *albeit *weakly to a first stimulation with iDCs loaded with either DSP or TMG peptides and significantly, not at all to WHK. Furthermore, when the cells were re-stimulated with iDCs primed with the corresponding peptides (DSP or TMG), much higher levels of IFN-γ were produced. Again, the response to the WHK peptide was weak (Fig. 3B). These results were reproducible in four separate experiments, in each case using cells from a different healthy donor and indicate that both the DSP and TMG peptides are capable of stimulating human lymphocytes to release IFN-γ, by a mechanism that requires DCs, but which appears to be independent of IL-12 production.

### DCs loaded with Hsp70 or Hsp70-PC from MDA-MB-231 cells can prime human lymphocytes to respond to MDA-MB-231 cell extracts

The consecutive stimulation of CD14- cells with DCs, as described above, was repeated except that different antigens pools were used in the first and second rounds of stimulation. Lymphocyte activation will only occur if the DCs present the same or a very similar antigen(s) in the two stimulations [39]. We first examined the consecutive stimulation of CD14^- ^cells with Hsp70-PCs and protein extracts from MDA-MB-231 cells.

**Figure 4:**
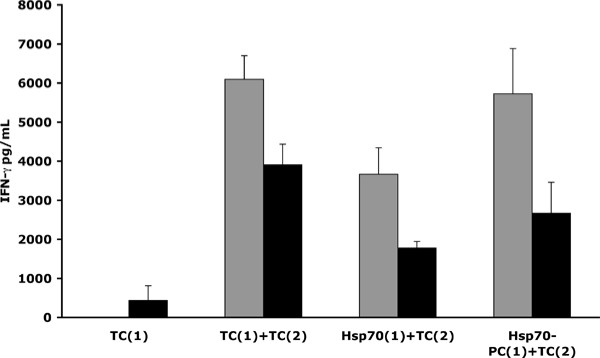
**iDCs loaded with Hsp70 or Hsp70-PC from MDA-MB-231 cells can prime human lymphocytes to respond to MDA-MB-231 cell extracts**. Purified Hsp70, Hsp70-PCs from MDA-MB-231 cells or soluble protein extracts from MDA-MB-231 tumour cells (TC) were incubated with iDCs isolated from two healthy female blood donors (Donor A; grey bars. Donor B; black bars). The iDCs were incubated [first stimulation (1)] with CD14^- ^PBMCs from the same donor. The cells were cultured for 10 days and then, re-incubated [second stimulation (2)] with iDCs from the same donor loaded with TC. Supernatants were tested for IFN-γ (pg/mL) production by ELISA. The label 'Hsp70-PC(1)/TC(2)' in this figure refers to IFN-γ production by CD14^-^PBMCs following first and second incubations with iDCs loaded with Hsp70-PC and TC respectively. Other labels follow a similar paradigm.

CD14^- ^PBMCs were incubated with irradiated autologous monocyte-derived DCs pulsed with Hsp70, Hsp70-PC or soluble total protein extracts from MDA-MB-231 cells. The cells were then cultured for 10 days in the presence of IL-2, following which they were re-stimulated with iDCs pulsed with soluble protein extracts from MDA-MB-231 cells. After a further 2 days incubation, supernatants were tested for IFN-γ production. As shown in Figure 4, CD14^- ^PBMCs, primed with Hsp70-pulsed iDCs and challenged with iDCs pulsed with MDA-MB-231 cell extracts (tumour cell: TC) in the second stimulation [Fig. 4 Hsp70(1)/TC(2)], produced IFN-γ (pg/mL) levels at 60% and 46% of that produced by cells primed and challenged with MDA-MB-231 total cell extracts [TC(1)+TC(2)], in two individual blood donors respectively. Significantly higher levels of IFN-γ were secreted when cells were primed with Hsp70-PC and challenged with iDCs pulsed with TC [Fig. 4 Hsp70-PC(1)/TC(2); 98% and 68% the levels produced by cells primed and challenged with MDA-MB-231 total cell extracts in the two donors respectively]. Little or no IFN-γ was released when CD14^- ^PBMCs received only a single exposure of iDCs pulsed with TC (Fig. 4; TC(1), nor when CD14^- ^PBMCs were incubated with iDCs stimulated with LPS alone for the first stimulation and challenged with iDCs loaded with the soluble cell extract (data not shown).

### DCs loaded with DSP or TMG peptides can prime human lymphocytes to respond to MDA-MB-231 cell extracts

**Figure 5:**
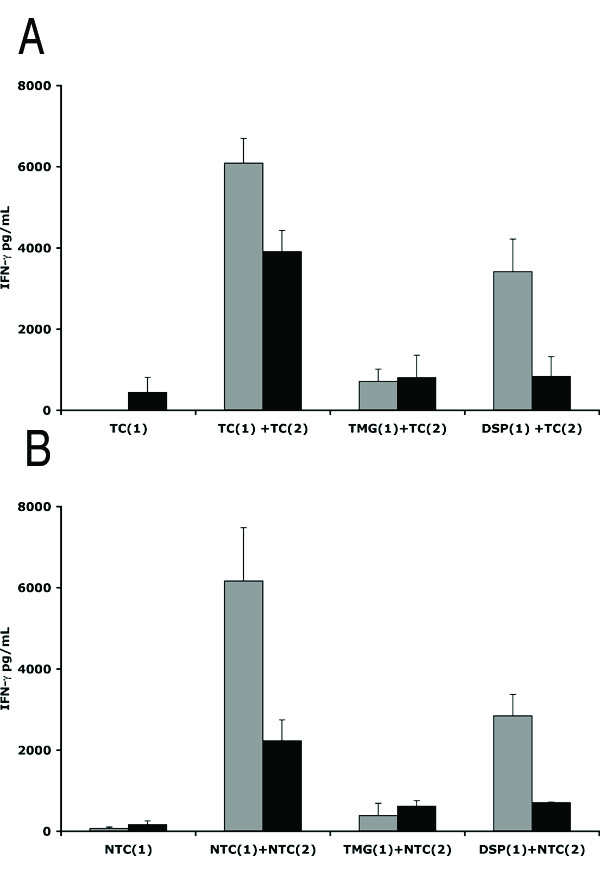
**iDCs loaded with DSP or TMG can prime human lymphocytes to respond to MDA-MB-231 cell extracts**. iDCsfrom two healthy female blood donors (Donor A; grey bars. Donor B; black bars), were incubated with the peptides DSP or TMG, or cell extracts from MDA-MB-231 tumour cells (TC) **[A] **or MCF-12A non-tumour cells (NTC) **[B]**. The loaded iDCs were incubated [First stimulation (1)] with CD14^- ^PBMCs from the same individual. The cells were cultured for 10 days and then, incubated for a second time [Second stimulation (2)] with iDCs loaded with either TC **[A] **or NTC **[B]**. Supernatants were assayed for IFN-γ (pg/mL) production by ELISA. The label 'DSP(1)/TC(2)' refers to IFN-γ production by CD14^-^PBMCs following first and second incubations with iDCs loaded with DSP peptide and TC respectively. Other labels follow a similar paradigm.

To determine if the mimic peptide DSP resembles any endogenous peptides or proteins present in the extracts from MDA-MB-231 tumour cells, the consecutive stimulation of CD14^- ^cells with different antigen pools was performed as described above. CD14^- ^PBMCs primed initially with iDCs loaded with the DSP peptide and subsequently stimulated with iDCs loaded with TC extracts secreted IFN-γ at 56% and 27% the levels produced when CD14^- ^cells were both primed and stimulated with TC extracts in donors A and B respectively, [Fig. 5A; DSP(1)+TC(2)]. Lower levels of IFN-γ were produced by CD14^- ^cells stimulated first with TMG and then with TC extracts [Fig. 5A; TMG(1)+TC(2)]. Little or no IFN-γ was detected when CD14^- ^PBMCs received only a single exposure to TC [Fig. 5A, TC(1)], nor was IFN-γ produced when PBMCs were incubated with iDCs stimulated by LPS alone in the first stimulation and then challenged with iDCs loaded with TCs in the second stimulation (data not shown).

To determine whether the T cell stimulation by the peptides was tumour cell-specific, the experiment was repeated but this time using a cell extract from a non-tumourigenic breast cell line, MCF12A (Fig. 5B non-tumour cells: NTC). CD14^- ^PBMCs first stimulated with iDCs loaded with the peptide DSP and then challenged with NTC total protein extract, produced IFN-γ at 46% and 32% the levels produced when CD14^- ^cells were both primed and stimulated with NTC total protein extract in donors A and B, respectively (Fig. 5B; DSP(1)+NTC(2)). In contrast, when primed with iDCs loaded with TMG and stimulated with iDCs loaded with NTC total protein extract, the relative IFN-γ levels were 6.3% and 28% in donors A and B respectively (Fig. 5B: TMG(1)+NTC(2)). There was little detectable IFN-γ produced by cells stimulated by a single exposure to NTC total protein extract [Fig. 5B, NTC(1)], nor when CD14^- ^cells were incubated with iDCs stimulated by LPS alone in the first stimulation and then challenged with iDCs loaded with NTCs in the second stimulation (data not shown).

### iDCs loaded with Hsp70 or Hsp70-PC can prime human lymphocytes to respond to DSP peptide

**Figure 6:**
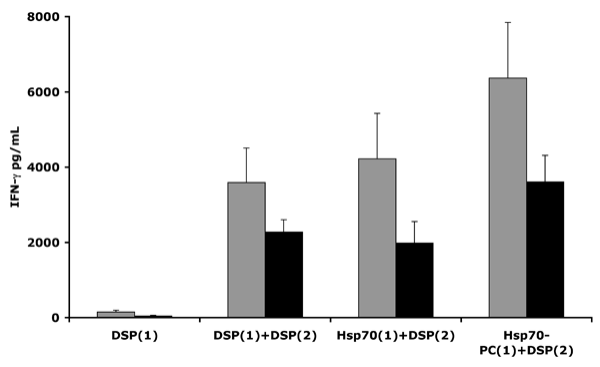
**iDCs loaded with Hsp70 or Hsp70-PC from MDA-MB-231 cells can prime human lymphocytes to respond to synthetic DSP peptide**. IDCs from two healthy female donors (Donor A; grey bars. Donor B; black bars), were incubated with either Hsp70 or Hsp70-PCs from MDA-MB-231 cells or the synthetic DSP peptide. The iDCs were then incubated with CD14^- ^PBMCs from the same donor. [First stimulation (1)]. The cells were cultured for 10 days and then, incubated with a second batch of iDCs loaded with the synthetic peptide DSP [Second stimulation (2)]. The supernatants were assayed for IFN-γ production (pg/mL). The label 'Hsp70-PC(1)/DSP(2)' refers to IFN-γ production by CD14^-^PBMCs following first and second stimulations with iDCs loaded with Hsp70-PC and the DSP peptide respectively. Other labels in the figure follow a similar paradigm.

CD14^- ^PBMCs were first stimulated with iDCs pulsed with the DSP peptide, Hsp70 or Hsp70-PCs and subsequently stimulated with iDCs pulsed with the mimic peptide DSP. As shown in Figure 6, CD14^- ^PBMCs from two individual donors, primed with Hsp70-PCs respond very effectively to a second stimulation by iDCs loaded with the DSP peptide [Hsp70-PC (1) + DSP (2)] relative to that observed when the DSP peptide is used in both the first and second stimulations [DSP (1) +DSP (2)]. When cells were stimulated with Hsp70, depleted of the associated peptides in the first stimulation and then challenged with iDCs loaded with the DSP peptide in the second stimulation, IFN-γ secretion is also observed however to a lesser degree than that observed with Hsp-70-PC [Hsp70 (1) +DSP (2)]. Reiterating the previous finding (Fig. 3A), a single stimulation of CD14^- ^PBMCs with the DSP peptide is not sufficient to elicit detectible IFN-γ production [DSP (1)], nor was IFN-γ produced if PBMCs were incubated with iDCs stimulated by LPS alone in the first stimulation and then challenged with iDCs loaded with the DSP peptide in the second stimulation (data not shown).

Taken together, our data suggest that the mimic peptide DSP resembles immunogenic peptides and/or protein components from both tumourigenic and non-tumourigenic breast cell lines and has the ability to stimulate human CD14^- ^PBMCs *in vitro*. Furthermore, the DSP peptide may structurally resemble peptides complexed with Hsp70.

## Discussion

Phage display libraries, since their discovery in 1990 [42], have been used to identify high affinity ligands to a variety of molecules small and large. Recently, Tiwari and colleagues [28] have used two different peptide phage display libraries to identify potential peptides that mimic the antibody-binding epitopes of the extracellular domain of HER-2/neu antigen. Employing anti-HER-2/neu monoclonal antibodies as a bait and four rounds of bio panning, the three selected peptides were able to elicit humoral immune responses in mice and to inhibit the binding of the bait to HER-2/neu [28] antigen, thus illustrating that synthetic peptide mimics can elicit immune stimulatory activities.

The primary aim of this study was to test a "proof of principle", that functional synthetic mimics of native Hsp70-PCs could be identified by using two step bio-panning of random peptide phage display libraries. Previous studies have shown that Hsp-PCs from mice and humans can induce specific T-cell responses [7,21]. The mimic peptides, in principle, should structurally resemble the Hsp70-PCs used as the initial bait and may have functional properties similar to Hsp70-PCs such as the ability to stimulate T-cells. The Hsp70-PCs preparation used in this study was obtained by affinity selection on an ADP-agarose column. Hsp70-PCs, isolated by this method, have been shown to contain a wide array of peptides and to possess immune-stimulatory activity [7,43]. Thus, the starting material for the bio-panning was the pool of peptides complexed with Hsp70. This approach circumvents the need for the purification of individual peptides from the Hsp70-PC fraction and selects for the abundant ones. Similar bio-panning approaches have proved successful in identifying tissue-specific peptide ligands [(25); see ref. [37] for a review].

A variety of phages displaying unique peptides were identified by the bio-panning method. There appeared to be a high degree of enrichment of specific sequences following four rounds of bio-panning; certain peptides selected were common to at least two bio-pannings and one of the peptides (TMG) was recovered in all three bio-pannings. The diversity of peptides identified may be in part due to the complexity of the Hsp70-PCs fraction used as the bait. The peptide TMG was selected in three independent bio-panning experiments, suggesting that the motif recognised by TMG may be consistently abundant in the three pools of Hsp70-PCs.

Three of the 'recogniser' peptides, identified in multiple bio-panning experiments, were then used in a reverse bio-panning experiment to identify sequences that interact with these peptides. Two of these recogniser peptides, NNY and IER, selected a wide variety of binders, one of which, SVS, was common between the two selected pools. Subsequent bio-panning experiments with an unrelated peptide bait, consistently selected the SVS peptide (Arnaiz, James and Bond unpublished data). Interestingly, this same phage peptide had been identified in two unrelated bio-pannings for peptides interacting with (i) murine cerebellar granular neurons and (ii), a Japanese encephalitis virus envelope protein neutralizing antibody [40,41]. The consistent selection of SVS with unrelated baits perhaps suggests that this peptide may recognise some common structure among all baits, for example the peptide bond backbone. Unlike the peptides NNY and IER, the TMG selected only a single displayed peptide after four rounds of bio-panning. Due to the high degree of selectivity of the TMG peptide and the fact that this peptide was identified in three independent bio-panning experiments, we focused our subsequent analysis on this TMG-DSP 'recogniser-mimic' pair.

The ability of the synthetic peptides to activate lymphocytes was examined using an *in vitro *assay. The results show that lymphocytes were stimulated but required two consecutive iDC-mediated exposures to either the mimic peptide DSP or the recogniser peptide TMG. Unlike LPS, these peptides did not stimulate iDCs to produce IL-12. Therefore, we can conclude that (a) the activation of lymphocytes must be dependent upon the uptake of the peptide by iDCs and its representation to lymphocytes and (b) it is not the result of any adjuvant-like contaminant present in the peptide preparation. The DSP peptide showed lymphocyte stimulatory activity while, another peptide WHK produced no stimulation. Surprisingly, we did observe lymphocyte stimulation with the recogniser peptide TMG. The reason why lymphocyte activation was not limited to only mimics is currently unclear. The observed differences in effectiveness between the peptides may be reflective of the different proteolytic processing and/or the preference of the different HLA class I molecules for presentation of 9-mer peptides with specific amino acids in anchor positions. A search of the comprehensive database SYFPEITHI [44] for HLA class I ligands with the peptide sequences revealed a higher likelihood for DSP and TMG than WHK to be presented by the HLA class I molecule (data not shown). Furthermore, based on our model of recognisers and mimics described above, it is quite possible that peptides structurally equivalent to recogniser peptides may also be present in the pool of Hsp70-PCs, for example, the EGF receptor (a possible 'recogniser' molecule) is activated by autocrine or paracrine growth factor loops and is known to be over-expressed in at least 50% of all epithelial malignancies [45] as is its ligand, EGF, (a possible 'mimic' molecule). Supporting this view, we find that both TMG and DSP peptides but not WHK specifically bind to MDA-MB-231 cells indicating that either peptide can interact with cellular components within these cells (Arnaiz and Bond, unpublished results).

To determine if the DSP peptide represents a mimic of true tumour antigens present in tumour cells and in particular tumour antigens in the Hsp70-PCs pool, we employed an assay in which lymphocytes are stimulated in two consecutive rounds with different antigen pools. We find that tumour cell extracts contain certain antigens in common with those present in the Hsp70-PCs fraction as they successfully stimulate T-cells previously primed with Hsp70-PCs from the same tumour cells. Additionally, we observe that Hsp70 alone, in the absence of any associated peptides, can prime T-cells to respond to tumour cell extracts. Thus Hsp70 in addition to chaperoning peptides into the antigen processing pathway of iDC, may also trigger IFN-γ production in a similar way to that of LPS [46-49]. These findings are in agreement with previous data showing that Hsp70 can enhance the ability of APCs for antigen uptake [50,51] and can activate T cells *in vitro *an *in vivo *[10,52]. Therefore, one could envisage a pool of antigens (peptides) being chaperoned by adjuvant molecules such as Hsp70 which can also facilitate their uptake by the APCs through Hsp-specific receptors (e.g., CD91) in the case of tissue damage or necrosis. These peptides may be then re-presented to T-cells, through the MHC class I antigen processing pathway (cross-priming). Thus, the reconstitution of peptides with Heat shock proteins such as Hsp70, might be an important strategy to ensure an enhancement of the T-cell response to peptides [16,53].

Using the same technique, we also show that the DSP peptide resembles antigens present in total cell extracts from either tumourigenic (MDA-MB-231) or non-tumourigenic (MCF-12A) breast carcinoma cell lines. Thus, the DSP peptide may mimic a common antigen in both cell lines. The TMG peptide showed lower levels of lymphocyte stimulation following a second exposure to either extracts from MDA-MB-231 or MCF-12A cell lines. We also show that lymphocytes incubated initially with iDCs loaded with Hsp70-PCs can be re-stimulated with iDCs loaded the DSP peptide, again suggesting that this peptide resembles antigenic peptides associated with the Hsp70 in these cell lines.

## Conclusion

In conclusion, we have developed a bio-panning approach to enrich from phage display libraries potential peptide mimics of Hsp70-PCs and an *in vitro *lymphocyte activation assay to validate their potential as tumour specific antigen mimics. Such peptides could be further modified or combined with other molecules to develop potential tumour vaccines. In this initial study we have identified two peptides with lymphocyte stimulatory activity. We envisage further adjustments to the bio-panning protocol such as pre-adsorbing the phage display library to a non-tumourigenic Hsp70-PC fraction to enrich for true tumour-specific recogniser peptides and/or the use of tandem mass spectroscopy to directly sequence and identify the peptides associated with Hsp70-PCs. In the latter case, their synthetic equivalents can be tested directly in T-cell stimulation. High throughput assays could be developed to relatively quickly screen large numbers of synthetic recogniser/mimic peptides identified through the strategies described here. Such mimics could be further modified to increase their immunological effectiveness either inherently at the sequence level or by using a cocktail of selected mimics for a given tumour based on their IFN-γ response. Furthermore, the essential concept of the screening strategy can be applied to many other potential drug and biomarker discovery applications.

## Declaration of competing interests

The author(s) declare that they have no competing interests.

## Authors' contributions

BA and L M-E carried out the in vitro immuno-stimulatory assays. ST and DD contributed to the design and supervision of the immuno-stimulatory assays. TCJ and UB conceived and designed the methods for the isolation of the peptide mimics of Hsp-PCs and identified all of the peptides described in the manuscript.
